# Undifferentiated high-grade sarcoma with UES-like features in the retroperitoneum: a Case Report with TP53 p.R306 mutation

**DOI:** 10.3389/fonc.2025.1672264

**Published:** 2025-10-06

**Authors:** Yinan Zhu, Mingfang Sun, Ziyue Wang, Xuyong Lin

**Affiliations:** Department of Pathology, the First Affiliated Hospital and College of Basic Medical Sciences, China Medical University, Shenyang, China

**Keywords:** high-grade sarcoma, polymorphic undifferentiated sarcoma, liver undifferentiated embryonal sarcoma, UESL, pathology

## Abstract

Undifferentiated embryonal sarcoma(UES) is a rare, highly malignant tumor mainly originating from the liver, composed of primitive undifferentiated mesenchymal cells. Undifferentiated embryonal sarcoma of the liver(UESL) is a rare entity. Here, we report a case of abdominal mass in a young female that morphologically resembles UES without liver involved. Notably, the mass was located from the abdominal cavity to the retroperitoneum without any correlation with the liver. The patient had no history of prior tumors, and detailed examinations of the liver revealed no definite lesions. In addition, we propose undifferentiated high-grade sarcoma with UES-like features in the retroperitoneum, in which TP53 gene mutation may serve as the initiating factor for tumorigenesis.

## 1 Introduction

Undifferentiated embryonal sarcoma of the liver (UESL) is a rare, highly malignant tumor originating from the liver, composed of primitive undifferentiated mesenchymal cells. With an overall incidence of 1 in 1 million, it represents the third most common hepatic malignant tumor in children, second only to hepatoblastoma and hepatocellular carcinoma, accounting for 9-15% of pediatric liver malignancies (1, 2). The incidence peak occurs at 6–10 years of age, while a minority of cases affect young to middle-aged adults and even the elderly. Among adult patients, females predominate (3). Here, we report a case of abdominal mass in a young female that morphologically resembles UESL. Notably, the mass was located from the abdominal cavity to the retroperitoneum without any correlation with the liver. The patient had no history of prior tumors, and detailed examinations of the liver revealed no definite lesions. We propose this as undifferentiated high-grade sarcoma with UES-like features in the retroperitoneum, in which TP53 gene mutation may serve as the initiating factor for tumorigenesis.

Our case report offers a detailed and comprehensive account of the clinical diagnosis, encompassing imaging findings, histological characteristics, and genetic features, thereby contributing to the advancement of clinical practice related to UES.

## 2 Case presentation

**Figure 1:**
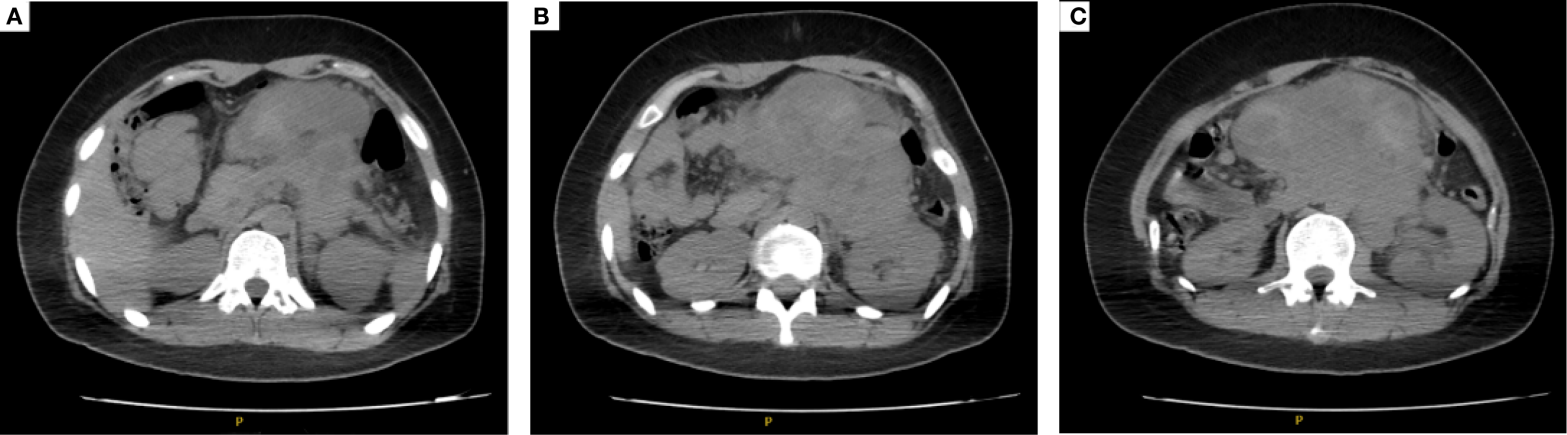
Plain abdominal CT showed a large irregular soft tissue mass in the abdominal cavity to retroperitoneum [not connected to the liver; **(A)**], measuring approximately 14cm × 8cm. The mass exhibited heterogeneous density with an average CT value of 18–62 Hu and a relatively clear boundary **(B)**. Adjacent structures were compressed, especially the left kidney, with dilatation of the left renal pelvis **(C)**.

A 22-year-old female patient presented with left lower abdominal distension for two months, which aggravated after meals. She had no history of hepatitis, liver cirrhosis, or tumors. Plain abdominal CT showed a large irregular soft tissue mass in the abdominal cavity to retroperitoneum (not connected to the liver; **Figure 1A**), measuring approximately 14 cm × 8 cm. The mass exhibited heterogeneous density with an average CT value of 18–62 Hu and a relatively clear boundary (**Figure 1B**). Adjacent structures were compressed, especially the left kidney, with dilatation of the left renal pelvis (**Figure 1C**). Enlarged lymph nodes were observed in the retroperitoneum. Laboratory tests revealed decreased hemoglobin concentration (107 g/L [115-150]), reduced serum prealbumin (10.3 mg/dL [18-35]), and elevated plasma fibrinogen (5.47 g/L [2-4]), while other test results were within normal ranges. The indicators of renal function, such as urea (3.73mmol/L[2.6-7.5]) and creatinine (44umol/L[41-73]), were not abnormal. Tumor markers (such as AFP, CEA, CA125) were all in the normal scope. After admission and completion of routine examinations, the patient underwent puncture biopsy of the abdominal mass.

**Figure 2:**
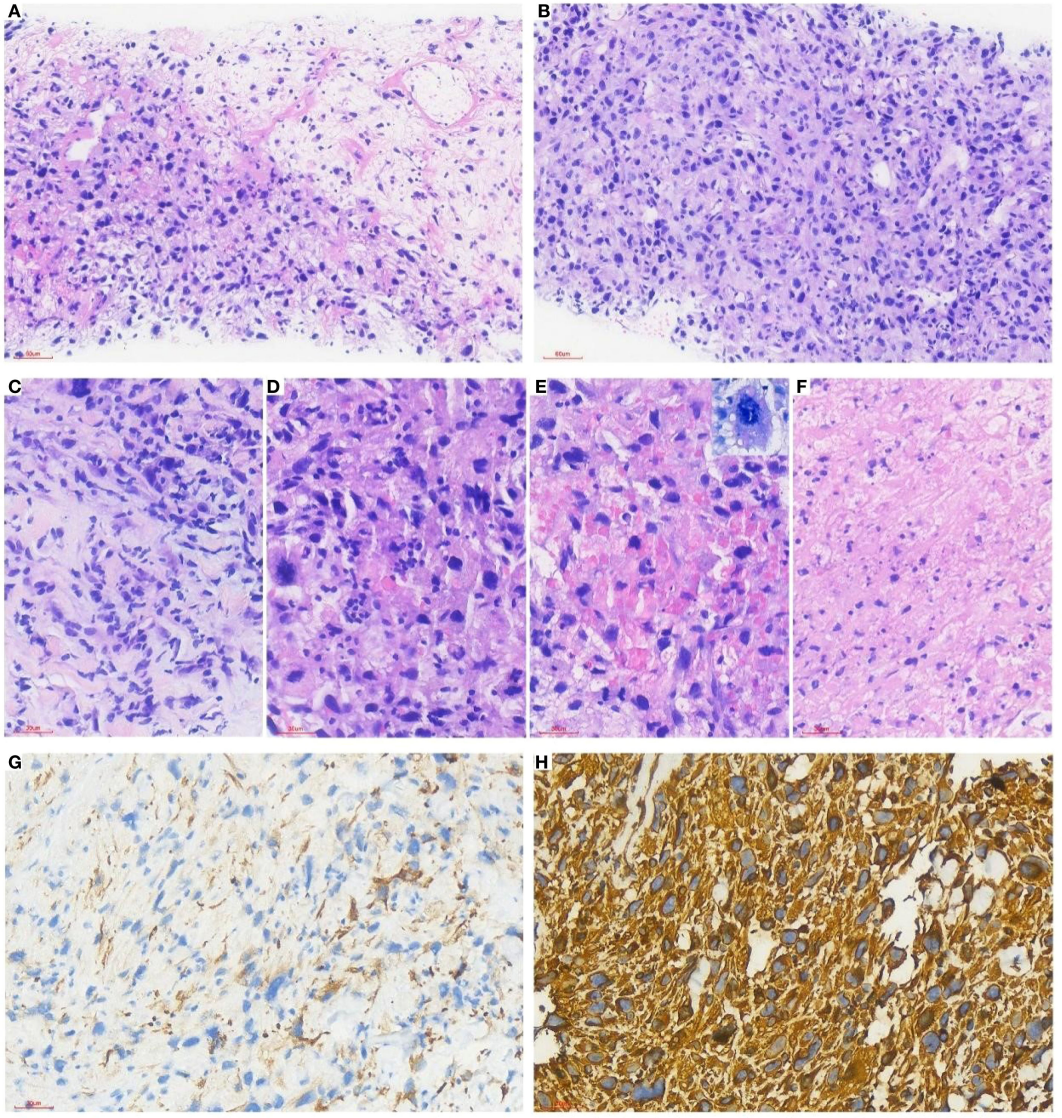
The tumor showed variable cellularity **(A)**, with abundant thin-walled blood vessels and myxoid stroma **(B, C)**. Tumor cells were spindle-shaped, oval, or irregular, exhibiting marked atypia, including deeply stained bizarre cells and multinucleated cells. Nucleoli were inconspicuous, and mitotic figures were readily observable. Eosinophilic globules of varying sizes (positive for PAS staining) were detected in the cytoplasm of tumor cells and extracellular matrix **(D, E)**, accompanied by focal tumor necrosis **(F)**. Focal positivity for CK was noted **(G)**, while Vimentin **(H)** and SMA were positive.

The tumor showed variable cellularity (**Figure 2A**), with abundant thin-walled blood vessels and myxoid stroma (**Figures 2B, C**). Tumor cells were spindle-shaped, oval, or irregular, exhibiting marked atypia, including deeply stained bizarre cells and multinucleated cells. Nucleoli were inconspicuous, and mitotic figures were readily observable. Eosinophilic globules of varying sizes (positive for PAS staining) were detected in the cytoplasm of tumor cells and extracellular matrix (**Figures 2D, E**), accompanied by focal tumor necrosis (**Figure 2F**).The results of immunohistochemical (IHC) were as follows: Focal positivity for CK was noted (**Figure 2G**), while Vimentin (**Figure 2H**) and SMA were positive. CD117 showed weak positivity in partial areas, whereas Desmin, MyoD1, Calretinin, CK5/6, S-100, CD34, Dog-1, and MDM2 were negative. P53 was wild-type, and the Ki-67 proliferation index was approximately 60%.DNA+RNA combined next-generation sequencing (NGS) revealed a TP53 p.R306* mutation, PDGFRA copy number amplification, missense mutations in ATR, FANCA, and FH, and a nonsense mutation in RREB1.Based on the morphology and immunohistochemical results, our diagnosis tended to be undifferentiated high-grade sarcoma with UES-like features.

With a confirmed diagnosis of malignant tumor, the patient did not receive subsequent treatment and died two months later. The patient ultimately died of multiple organ failure caused by rapid tumor progression.

## 3 Discussion

This is a case of an abdominal mass in a young female patient, which showed no definite correlation with abdominal viscera. Histologically, it was characterized by disorganized diffuse distribution of highly atypical cells with variable density, accompanied by numerous pathological mitotic figures and necrosis, exhibiting morphological features of high-grade sarcoma. Therefore, extensive immunohistochemical staining was performed to assist in the diagnosis. The immunohistochemistry of this case lacked specific immunophenotype. Tumor cells showed diffuse positive expression of vimentin and SMA, with partial expression of broad-spectrum CK and CD117. Although SMA was diffusely positive, negative staining for Desmin and MyoD1 excluded myogenic tumors. Additionally, CD117 showed cytoplasmic positivity in partial areas, but negative CD34 and Dog-1 expression did not support the diagnosis of gastrointestinal stromal tumor. Positive BAP1 and negative Calretinin/CK5/6 were inconsistent with the immunophenotype of sarcomatoid mesothelioma. Negative S-100 staining excluded neurogenic tumors, melanoma, and adipogenic tumors. With markedly atypical spindle cells and pleomorphic cells arranged in a disorganized manner, frequent bizarre multinucleated tumor giant cells, and lack of specific immunohistochemical markers, is pleomorphic undifferentiated sarcoma an appropriate diagnosis? Upon careful observation of HE sections, we found eosinophilic globules of varying sizes in the cytoplasm of partial cells and stroma, which were positive for PAS staining. This morphology is highly similar to that of undifferentiated embryonal sarcoma (UESL).

Undifferentiated embryonal sarcoma of the liver (UESL) almost exclusively occurs in the liver, typically presenting as a single, well-demarcated large nodule in the right hepatic lobe, composed of solid and cystic components. Extrahepatic dissemination is observed in 5-15% of patients (3–5). The diagnosis lacks specific clinical manifestations, laboratory markers, and imaging features, with definitive diagnosis primarily relying on histological characteristics and immunophenotype. Histologically, UESL is characterized by highly atypical sarcomatoid cells with variable density embedded in a myxoid stroma. Tumor cells are spindle-shaped or stellate, with indistinct boundaries, inconspicuous nucleoli, and readily apparent mitotic figures. Deeply stained pleomorphic tumor giant cells and multinucleated giant cells are commonly seen, accompanied by hemorrhage and necrosis. A characteristic morphological feature of UESL is the presence of eosinophilic bodies of varying sizes within or outside tumor cell cytoplasm, which are positive for PAS staining and resistant to amylase digestion. Immunohistochemistry is nonspecific for evaluating the histogenesis of UESL, and individual markers are insufficient to distinguish UESL. Thus, extensive immunohistochemical staining is usually required for diagnosis, with negative markers playing a crucial role in excluding differential diagnoses (3, 6). The specific pathogenesis of UESL remains unclear, but its mesenchymal origin is widely accepted. Ultrastructural analysis shows that UESL tumor cells exhibit characteristics of undifferentiated cells, fibroblasts, myofibroblasts, smooth muscle, and skeletal muscle cells in a composite manner (6, 7). Some UESL cases may arise from malignant transformation of hepatic mesenchymal hamartoma (HMH), both involving translocation of the long arm of chromosome 19 (19q13.4) (6). Additionally, TP53 gene mutations and somatic mutations in WDR25, CMTM1, and DNAH17 may serve as driving factors for UESL (8–10).For the first time, Shimagaki T et al. (11) used genomic analysis to reveal 11 somatic cell mutations, including TP53 (R196*) and STK11 (F354L). This discovery enriched the understanding of UESL at the genomic level. However, these findings are based on analyses of a minority of cases, and experimental validation via genome-wide molecular analysis in large cohorts is still lacking.

Meanwhile, inconsistencies between IHC and genetic testing results were also observed in our cases. Furthermore, upon thorough review of previous literature, similar inconsistencies between IHC and genetic testing have been reported (12–14). We have summarized the potential causes for such discrepancies, which fall into two main scenarios. The first scenario: No loss of expression detected by IHC, while genomic variations identified by genetic testing. This discrepancy can be attributed to two key factors. On the one hand, tumors exhibit heterogeneous expression regions. Even tumors with positive protein expression (detected by IHC) may still harbor gene mutations and methylation events (15). On the other hand, missense mutations in protein-coding genes can lead to abnormal protein function without altering the protein’s antigenic structure (20). In such cases, IHC can still detect protein expression (as the antigenic structure remains intact), despite the presence of functional abnormalities, whereas next-generation sequencing (NGS) confirms the underlying genetic variation. The second scenario: Loss of expression detected by IHC, yet no genomic variations identified by NGS. We have summarized the reasons for this scenario. Firstly, presence of genes with functional compensation mechanisms may plays an important role in the process (16). IHC typically targets only one common specific protein, however, other genes may provide functional compensation. This can result in loss of the target protein’s expression (detected by IHC) without corresponding alterations in the gene itself. Secondly, pay attention to prior treatment history in patients, particularly neoadjuvant chemotherapy. Neoadjuvant chemoradiotherapy is known to interfere with protein expression, which may lead to this inconsistency (17).In addition, methylation of gene promoters is a possible factor. We hypothesize that methylation of gene promoters can suppress protein expression (resulting in IHC-negative findings) without causing structural changes to the gene (thus showing no variations on NGS).

Therefore, although the patient lacked hepatic lesions, combined with morphological and immunohistochemical findings, undifferentiated embryonal sarcoma (UES) was considered an appropriate diagnosis. Molecular pathology of this case suggested that the TP53 p.R306* mutation might serve as a driving factor for tumorigenesis. The mutation abundance of TP53 p.R306* was 44.44%, indicating a potential germline mutation. Regrettably, the patient did not receive follow-up treatment after diagnosis and succumbed to the tumor within a short period. As a result, no effective treatment experience could be derived from this case. Surgical resection and postoperative chemotherapy have been shown to be effective in the literature (18, 19).

## 4 Conclusion

In summary, we report a case of undifferentiated high-grade sarcoma with UES-like features in the retroperitoneum. The patient had no history of prior hepatic tumors or lesions in other sites, and imaging examinations excluded the possibility of hepatic involvement. We propose that this represents an undifferentiated embryonal sarcoma without liver involved, sharing similar morphological, immunohistochemical, and molecular characteristics with undifferentiated embryonal sarcoma of the liver (UESL). This case further expands the disease spectrum of such tumors.

## Data Availability

The original contributions presented in the study are included in the article/supplementary material. Further inquiries can be directed to the corresponding author/s.
